# Index Catalogue of the Library of the Surgeon-General’s Office, United States Army

**Published:** 1891-11

**Authors:** 


Index-Catalogue of the Library of the Surgeon-General's Office, United States Army. Authors and Subjects. Vol. XII. Reger-Shuttleworth. Quarto, pp. 1004. Compiled by John S. Billings, Surgeon United States Army. Washington: Government Printing Office. 1891.

This twelfth volume of the Index-Catalogue of the Surgeon-General's Office brings the work down to and including Reger-Shuttleworth. It contains 20,215 author's titles, representing 8,022 volumes and 18,090 pamphlets. It also includes 6,603 subject-titles of separate books and pamphlets, and 18,956 titles of articles in periodicals. This gives a total, up to date, in the twelve volumes of 137,578 author-titles, representing 66,855 volumes and 120,000 pamphlets; also 128,284 subject-titles of separate books and pamphlets, 393,808 titles of articles and periodicals, and 4,335 portraits. This easily makes it the most complete index-catalogue of any library in the world—and the end is not yet.

It is a wonderful collection of medical literature, and when we reflect that it is open to the use of the medical profession of America, we can but feel that each one of us has a special interest in this library. It is expected to complete the catalogue within the next three years, but, of course, much material has accumulated since this work was begun, hence the necessity for the issuance of additional volumes. It is further expected that this work will be continued as fast as the literature accumulates, so that practically the Index will become an annual. The medical profession of America owes the compiler a debt of gratitude for his great labor.

